# *O*-GlcNAcylation: The Underestimated Emerging Regulators of Skeletal Muscle Physiology

**DOI:** 10.3390/cells11111789

**Published:** 2022-05-30

**Authors:** Yang Liu, Ya-Jie Hu, Wen-Xuan Fan, Xin Quan, Bin Xu, Shi-Ze Li

**Affiliations:** National Experimental Teaching Demonstration Center of Animal Medicine Foundation, College of Animal Science and Veterinary Medicine, Heilongjiang Bayi Agricultural University, Daqing 163319, China; lymissive@163.com (Y.L.); huyajiess@163.com (Y.-J.H.); 15945687925@163.com (W.-X.F.); 13359612081@163.com (X.Q.)

**Keywords:** *O*-GlcNAc, skeletal muscle, metabolism, insulin resistance, sarcomere contraction and structure

## Abstract

*O*-GlcNAcylation is a highly dynamic, reversible and atypical glycosylation that regulates the activity, biological function, stability, sublocation and interaction of target proteins. *O*-GlcNAcylation receives and coordinates different signal inputs as an intracellular integrator similar to the nutrient sensor and stress receptor, which target multiple substrates with spatio-temporal analysis specifically to maintain cellular homeostasis and normal physiological functions. Our review gives a brief description of *O*-GlcNAcylation and its only two processing enzymes and HBP flux, which will help to better understand its physiological characteristics of sensing nutrition and environmental cues. This nutritional and stress-sensitive properties of *O*-GlcNAcylation allow it to participate in the precise regulation of skeletal muscle metabolism. This review discusses the mechanism of *O*-GlcNAcylation to alleviate metabolic disorders and the controversy about the insulin resistance of skeletal muscle. The level of global *O*-GlcNAcylation is precisely controlled and maintained in the “optimal zone”, and its abnormal changes is a potential factor in the pathogenesis of cancer, neurodegeneration, diabetes and diabetic complications. Although the essential role of *O*-GlcNAcylation in skeletal muscle physiology has been widely studied and recognized, it still is underestimated and overlooked. This review highlights the latest progress and potential mechanisms of *O*-GlcNAcylation in the regulation of skeletal muscle contraction and structural properties.

## 1. Introduction

The proteome is constantly changing and finding harmony with the needs of the organism and its cells, and various PTMs play a unique physiological function in these processes [1,2,3]. Glycosylation, the most extensive and diverse forms of PTMs in eukaryotic cells, contains different types of glycosylation pathways, involves complex metabolic networks and greatly amplifies the proteome by producing the multiple protein forms to instruct a myriad of functions [4,5]. *O*-GlcNAcylation is a dynamic, reversible and atypical glycosylation [6]. As its full name *O*-linked β-*N*-acetylglucosamine modification indicates, it involves the binding of a single GlcNAc to the serine and threonine residues of the target protein via a β-configuration *O*-glycosidic bond [7]. The precise dynamic homeostasis of *O*-GlcNAcylation is accurately completed only by OGT and OGA [8]. UDPGlcNAc is the only donor substrate of *O*-GlcNAcylation, which comes from HBP and responds to carbohydrate metabolism, fatty acid metabolism, protein metabolism and nucleotide metabolism [9,10]. It is worth noting that *O*-GlcNAcylation is also unique from other classical glycosylation in other aspects [11], and the enzymatic mechanism, amino acid residue sequence and conformation involved in *O*-GlcNAcylation are shown in Table S1.

**Figure 1:**
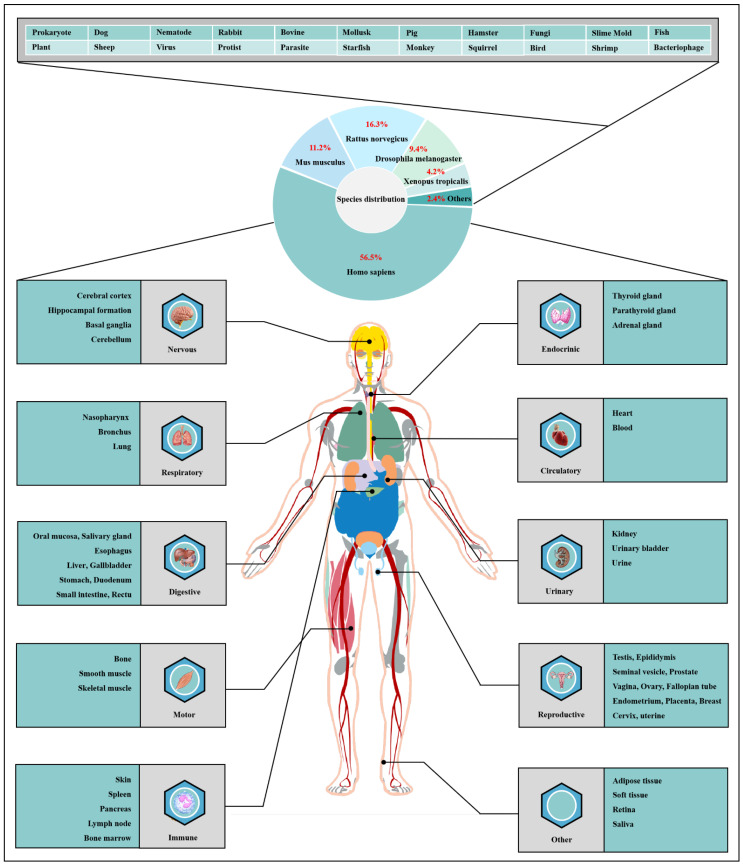
Species distribution of *O*-GlcNAcylation and its tissues’ distribution in *Homo sapiens*. The universality and conservation of *O*-GlcNAcylation is self-evident in filamentous fungi, worms, insects, plants and humans. There are more reports on the *O*-GlcNAcylation in human, mouse or rat, fruit fly and Caenorhabditis elegans species. However, *O*-GlcNAcylation has not been identified in yeast, and its similar role may be replaced by the *O*-mannosylation of nucleocytoplasmic proteins in yeast. *O*-Glcnacylation was found to occur in almost all major organs of *Homo sapiens*. This suggests that *O*-GlcNAcylation is essential for the survival of metazoans and is the root cause of the lethality of OGT and OGA knockout. Tissue distribution analysis emphasizes the strong characteristics of *O*-GlcNAcylationn in the brain and liver of *Homo sapiens*. Some organs or tissues with less distribution of *O*-GlcNAcylation may be limited by their own characteristics of less proteins.

**Figure 2:**
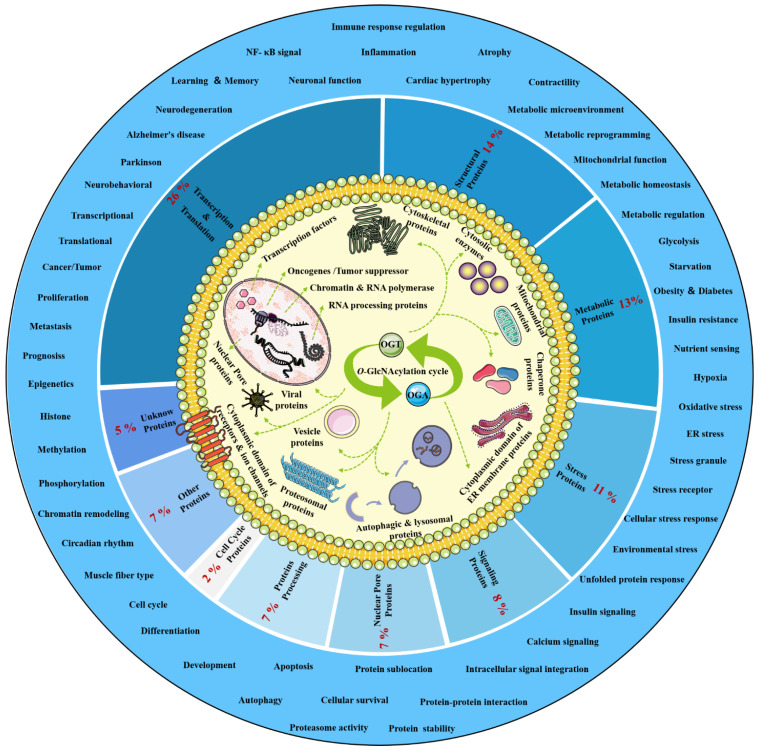
Classification, cellular distribution and physiological process of *O*-GlcNAcylated proteins. Studies on *O*-GlcNAcylation have been flourishing since its discovery. To date, a total of 5072 *O*-GlcNAcylated proteins have been identified by various techniques, and 1803 of these proteins contain 7002 different *O*-GlcNAcylation sites. However, such a large number may still be only a portion of the abundant dynamically modified proteins within the cellular compartments. The universality and conservation of *O*-GlcNAcylation is self-evident in filamentous fungi, worms, insects, plants and humans. There are more reports on the *O*-GlcNAcylation in human, mouse or rat, fruit fly and Caenorhabditis elegans species. However, *O*-GlcNAcylation has not been identified in yeast, and its similar role may be replaced by the *O*-mannosylation of nucleocytoplasmic proteins in yeast. These *O*-GlcNAcylated proteins occur in almost all cellular compartments. *O*-GlcNAcylated proteins are mainly located in the nuclear and cytoplasmic compartments of all metazoans and their infected viruses. Therefore, *O*-GlcNAcylation is considered to be one of the most abundant PTMs in the nucleocytoplasmic compartment. Secondly, some mitochondrial proteins are also *O*-GlcNAcylated. In addition, cytosolic domains of membrane proteins are also *O*-GlcNAcylated, as well as proteins involved in autophagy and proteosomal degradation of proteins, chaperone proteins, vesicle proteins and numerous cytosolic proteins and enzymes. Meanwhile, the distribution characteristics of *O*-GlcNAcylation also mean that almost all functions of proteins in regulating various cellular processes are covered. In other words, all functional classes of proteins are affected by *O*-GlcNAcylation, and these *O*-GlcNAcylated proteins are distributed according to protein function grouping, as shown above. Some of the largest classes of proteins include those in regulating metabolism, transcription and translation as well as structural proteins. Therefore, *O*-GlcNAcylation is involved in many cellular processes and pathology, including signal transduction, transcription, translation, chromatin remodeling, protein sublocation and stability, mitochondrial function and cell survival, etc.

*O*-GlcNAcylation exists in almost all organisms and is extremely conserved in filamentous fungi, worms, insects, plants and humans [12]. *O*-GlcNAcylation is also found in all major human organs, even in saliva and urine [13]. *O*-GlcNAcylation is abundant in the brain, liver, pancreas, skeletal muscle, adipose tissue and other organs and tissues, and plays an essential regulatory role in their physiology and pathology [7,14]. The species distribution of *O*-GlcNAcylation and its tissues distribution in *Homo sapiens* are illustrated in Figure 1. *O*-GlcNAcylation is present in almost all cellular compartments, such as the nucleus, cytoplasm, cytomembrane and mitochondria [15,16]. The distribution characteristics of *O*-GlcNAcylation also mean that almost all functions of proteins in regulating various cellular processes are covered [17]. *O*-GlcNAcylated proteins are grouped by protein function as shown in Figure 2. In the past 40 years since *O*-GlcNAcylation was first discovered, the *O*-GlcNAcylation of protein has been deeply understood and fruitful results have been obtained. Nearly 5000 human proteins and more than 7000 *O*-GlcNAcylated sites have been identified in thousands of related research studies [13,18]. *O*-GlcNAcylation affects the activity, stability, sublocation and biological function of target proteins. The abundance and cycle time scale of *O*-GlcNAcylation are very similar to that of phosphorylation [19]. Indeed, *O*-GlcNAcylation has surprisingly extensive crosstalk and forms a yin–yang relationship with phosphorylation, as do acetylation, ubiquitination and other PTMs [20,21]. Crosstalk between *O*-GlcNAcylation and these PTMs is shown in Box 1. *O*-GlcNAcylation receives and integrates metabolic signal pathway inputs from different partners to perceive external environmental disturbance, and ultimately induces adaptive molecular and physiological responses by targeting multiple substrates with the time-space specificity [22,23]. This physiological property makes *O*-GlcNAcylation extremely sensitive to nutrient availability and environmental changes and to become a nutrient sensor and stress receptor, thus participating in many biological processes [24]. For instance, *O*-GlcNAcylation plays a cytoprotective role under adverse conditions such as cold stress and oxidative stress [25,26,27]. *O*-GlcNAcylation helps to ensure the normal physiological function of cells by regulating a series of cellular processes, such as signal transduction [28,29], transcription [19,30], translation [31,32], autophagy [33,34], apoptosis [35], immune response [36,37,38], inflammation [39], chromatin remodeling [40] and metabolic reprogramming [41]. The destruction of the homeostasis of *O*-GlcNAcylation is closely related to the pathogenesis and progression of multiple diseases [42], such as multiple cancers [43,44,45], X-linked intellectual disability [46], neurodegenerative diseases [47,48], obesity [49], cardiovascular diseases [50,51], aging [52], diabetes and diabetes’ complications [53,54].

Box 1 **Extensive crosstalk between *O*-GlcNAcylation and other PTMs.**

More than 400 different types of PTMs individually or mutually regulate almost all aspects of protein function. Most proteins are heavily modified and the combination of various PTMs on a certain protein has explosively expanded its functional range. These PTMs often overlap the same domain on a certain protein and response to various cellular physiological states, and recruit other proteins to bind or regulate their activity. Therefore, the PTM code hypothesis has been proposed. This holds that the combination of multiple PTMs on a given protein produces a dynamic and specific "code" that is "read" and "translated" by the cell to drive complex biological outcomes. The corresponding enzymes involved in PTMs edit the code as editors and erasers to directly regulate protein function or indirectly regulate protein complex formation and signal transduction. In the past few years, innovations in PTMs detection methods have led to a rapid increase in proteomic data. However, the complexity of PTMs and the superficial understanding of thire biological function have hindered the development of this hypothesis. The *O*-GlcNAcylation plays a unique and important role in the PTM code due to its high sensitivity to cellular environmental and metabolic cues and extensive interactions with other PTMs. This will briefly discuss the crosstalk between *O*-GlcNAcylation and some common PTMs. The emphasis is on the interaction between *O*-GlcNAcylation and phosphorylation, and the interaction of *O*-GlcNAcylation with other PTMs is less well studied.

**1. Phosphorylation**

The intermodulation of *O*-GlcNAcylation and phosphorylation has attracted the most attention. *O*-GlcNAcylation occurs at Ser and Thr residues, which are also potential phosphorylation sites. However, phosphorylation at Tyr residues and *O*-GlcNAcylation also interact, even this regulation is even more efficient. There are currently four accepted forms of intermodulation between them. That is, the competitive modification at the same site, the alternating modification at different sites, the respective modification at different sites in adjacent regions, and the site-dependent alternating or simultaneous modification. *O*-GlcNAcylation and phosphorylation at the same or adjacent sites that do not occur simultaneously show negative interactions in a similar "on-off" pattern. Some proteins are simultaneously *O*-GlcNAcylated and phosphorylated at different sites. In this situation, *O*-GlcNAcylation often strongly inhibits phosphorylation in specific motifs that have been identified, and vice versa. However, there are also a few examples of *O*-GlcNAcylation enhanced phosphorylation and their physiological function. Thus, it can be seen that competition and dependence coexist between *O*-GlcNAcylation and phosphorylation.

In addition to the above way that *O*-GlcNAcylation directly acts on phosphorylated proteins, *O*-GlcNAcylation also interacts with phosphorylation by modifying phosphorylation related kinases. OGT and OGA form complexes with kinase and phosphatase. More than 100 kinases have been shown to contain identified *O*-GlcNAcylation sites, emphasizing the role of *O*-GlcNAcylation in directly regulating kinase function. A specific suitable example of complex regulation between kinase and *O*-GlcNAcylation is CaMKIV. CaMKIV contained at least five *O*-GlcNAcyclated sites. CaMKIV is activated by the phosphorylation at Thr^200^ and the displacement of PP2A. During the activation process, the increase of interaction between CaMKIV and OGA during activation led to the rapid decrease of *O*-GlcNAcylation level. Inactivated CaMKIV recruited OGT to restore *O*-GlcNAcylation. CaMKIV also activate OGT by promoting the phosphorylation of OGT. AMPK is another interesting example and is regulated by its own *O*-GlcNAcylation. AMPK phosphorylates GFAT at Ser^243^, resulting in decreased GFAT activity to reduce *O*-GlcNAcylation levels. AMPK phosphorylates OGT at Thr^444^, which leads to the changes in cell localization and substrate specificity of OGT. *O*-GlcNAcylation of PTP1B at Ser^104^, Ser^201^ and Ser^386^ increases its enzyme activity, but other phosphatase studies were few.

On balance, *O*-GlcNAcylation and phosphorylation form a highly dynamic balance and cooperate to complete a variety of complex biological processes, which is widely known as the "Yin-Yang" hypothesis. However, cross dialogue between *O*-GlcNAcylation and phosphorylation are complex at present. Therefore, it is still impossible to predict in advance how they will interact on a single protein.

**2. Acetylation**

*O*-GlcNAcylation of Rela/p65 at Thr^305^ and Thr^315^ promotes its acetylation at Lys^310^, which is necessary for the full transcriptional activity of Rela/p65. Meanwhile, HDAC4 has been shown to be modified by *O*-GlcNAcylation at Ser^642^, further demonstrating the direct interaction between *O*-GlcNAcylation and acetylation. Crosstalk between *O*-GlcNAcylation and acetylation is also involved in epigenetic regulation. For example, ManNAc induces switching from the inactive state by *Ogt-Sirt1* to the active state by *Oga*, *p300*, and *CBP* at the *Hcrt* gene locus. The histone acetyltransferase domain of OGA also shows that they have an intrinsic reciprocal relationship. SIN3A interacts directly with OGT, which is a histone deacetylase and transcription corepressor. In addition, OGT and OGA are also acetylated, but the effect on their function needs to be further confirmed.

**3. Ubiquitylation**

*O*-GlcNAcylation of target proteins prevents their ubiquitination to enhances their own stability. The potential mechanism may be the crosstalk between *O*-GlcNAcylated protein and phosphorylation to indirectly regulate ubiquitination or directly regulate ubiquitination by recruiting deubiquitylases. *O*-GlcNAcylation of histone H2B at Ser^112^ promotes its K120 monoubiquitination to transcriptional activation by using GlcNAc as an anchor of ubiquitin ligase. OGT and OGA are ubiquitinated, but the detailed impact and mechanism need to be further clarified.

**4. Methylation**

*O*-GlcNAcylation of histone methyltransferase enhancer of EZH2 at Ser*75* enhances its stability to promote the formation of H3K27me3, which helps to regulate gene expression related to skeletal muscle insulin sensitivity, tumor inhibition and neuronal memory formation. TET promotes DNA demethylation by hydroxylating 5mC. It was found that OGT interacted with TET protein to regulate transcription. This interaction provides a link between DNA modification, methylation and *O*-GlcNAcylation. The effect of this interaction on gene expression varies depending on TET family members and the environment.

Many studies have found that fast and slow skeletal muscles have different characteristics of *O*-GlcNAcylation during rest, exercise or muscle atrophy [55,56,57,58]. Recent data also show that *O*-GlcNAcylation regulates energy metabolism [59], mediates insulin resistance [59,60] and participates in different physiological processes of skeletal muscle [61,62]. Meanwhile, *O*-GlcNAcylation plays a potential role in many diseases related to skeletal muscle defects, such as neuromuscular diseases and amyotrophy [63,64,65]. Moreover, a great quantity of *O*-GlcNAcylated contractile and structural proteins were identified in sarcomeres, such as actin and myosin [66,67]. *O*-GlcNAcylation is a physiological mediator of skeletal muscle, such as contractile and structural properties, and myocardial and smooth muscle are no exception [68,69,70,71]. However, the effect of *O*-GlcNAcylation on skeletal muscle is still underestimated. Therefore, this review mainly explores the role and potential physiological mechanism of OGT in mediating skeletal muscle metabolism, regulating skeletal muscle contraction and maintaining the basic structure of skeletal muscle.

## 2. Dynamic *O*-GlcNAcylation Cycle and Hexosamine Biosynthesis Pathway

### 2.1. OGT and OGA Are the Only Antagonistic Enzymes for Precisely Regulating the O-GlcNAcylation Cycle in Space-Time Specificity

Unlike the complex regulation of other PTMs, the *O*-GlcNAcylation cycle of thousands of proteins is regulated by the synergistic action of OGT and OGA alone [72]. OGT transfers GlcNAc from UDP-GlcNAc to the hydroxyl groups in the threonine and serine residues of the target protein [73]. In contrast, OGA hydrolyzes GlcNAc from the *O*-GlcNAcylated protein [74]. The biological properties of OGT and OGA allow for the addition and removal of reversible GlcNAc multiple times quickly during theprotein lifetime to produce high kinetics of *O*-GlcNAcylation. OGT and OGA recognition and prompt mechanisms have always been the focus of OGT research. However, *Ogt* knockout is embryo lethal, which hinders the understanding of the precise biological functions of OGT and OGA [75]. This research was followed by the rapid development of highly effective and specific OGT and OGA inhibitors [76]. In addition, techniques and tools, such as purification and identification of *O*-GlcNAcylation, have greatly improved over the past few decades [77]. Here, we summarize the tools, tactics and objectives commonly used in *O*-GlcNAcylation research in Table S2, hoping to provide a basis for innovative solutions to overcome new challenges.

**Figure 3:**
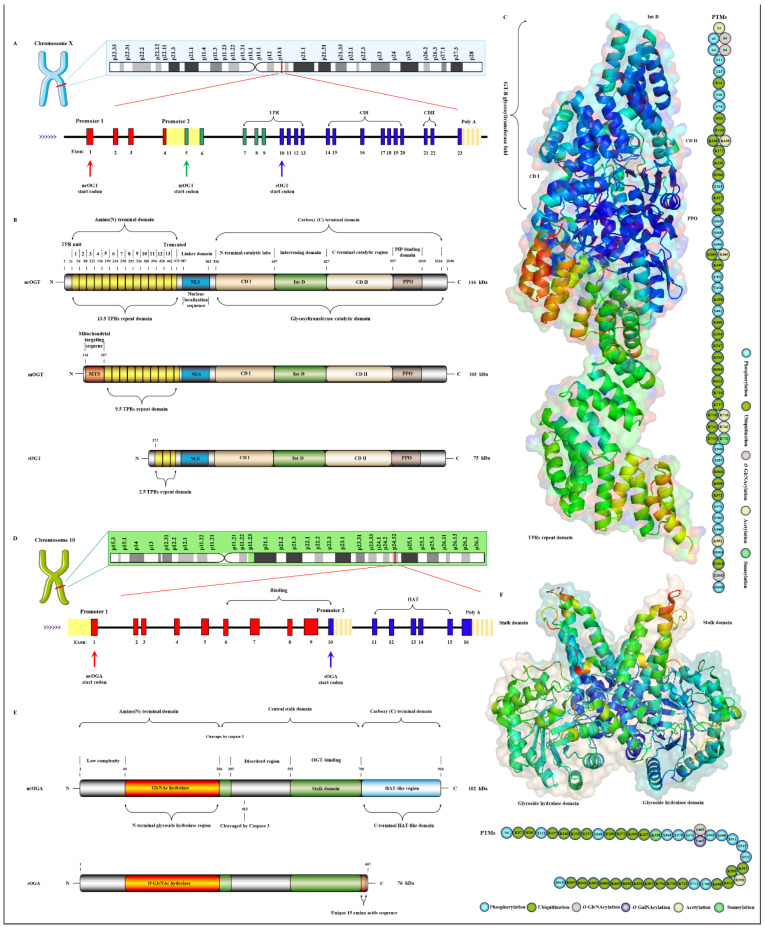
Genomic and proteomic structures of various subtypes of human OGT and OGA. (**A**) Human *Ogt* gene mapping and structure. (**B**) Primary protein structure of three subforms of human OGT. Subtype. (**C**) Advanced structures of human OGT in 3D and its PTMs. The advanced structure of OGA is displayed in cartoon and surface form with 5M7R in the protein data bank by PyMOL Molecular Graphics System, v2.5.2 (Schrödinger, LLC, New York, NY, USA). The various post-translational modification sites of OGT are present. These predicted modification sites are derived from the PhosphoSitePlus database (https://www.phosphosite.org, (accessed on 10 March 2022). (**D**) Human *Oga* gene mapping and structure. (**E**) Primary protein structure of three subforms of human OGA. Subtype. (**F**) Advanced structures of human OGA in 3D and its PTMs.

OGT has been found to exist in many organisms with high homology. Here, three subtypes of human OGT are taken as an example to demonstrate its gene and protein structure characteristics. OGT has only three isoforms: ncOGT, mimOGT and sOGT [78]. The genomic structure of human OGT is shown in Figure 3A. *Ogt* is a single gene residing on the chromosome X (Xq13.1) [79]. X inactivation regulates *Ogt* transcription in female mammals, and Xq13.1 is a region involved in Parkinson’s pathology [80]. *Ogt* contains 23 exons with alternatively spliced variants [81]. Exon 1 and promoter 1 produce ncOGT. Promoter 2 and the alternative start codon in exon 5 produce mOGT. The alternative start codon in exon 10 generates sOGT. All subtypes of OGT are mainly composed of the amino-terminal TPR domain, the same central linker domain and carboxyl-terminal catalytic domain [82]. All subtypes of OGT are shown in Figure 3B. The three subtypes of OGT contain different numbers of TPRs: 13.5 TPRs in nOGT, 9.5 TPRs in mOGT and 2.5 TPRs in sOGT [83]. Each TPR unit is comprised of 34 amino acid motifs. These TRP units are α-helical and clustered to form a repeating antiparallel helix-turn-helix super spiral [84]. TPRs acts like a “gatekeeper” to identify and interact with the target substrates by contacting their side chains [85]. The mutation of conserved aspartate residues in TPRs has resulted in significant changes in the selectivity, preference and *O*-GlcNAcylated rate of OGT for target substrates [86]. The influence of TPRs on substrate preference may also be the potential reason for their different cellular localization: mOGT exists in the mitochondria, while ncOGT and sOGT exist in the cytoplasm and nucleus, respectively [87]. In addition, TPRs also has a certain effect on the activity of OGT, and this effect can be exerted only by partial TPRs [88]. TPRs deleting the front 3 or 6 units do not affect the glycosylation of substrates by OGT; however, TPRs deleting the front 9 or 11 units will deactivate OGT [83,89]. OGT belongs to the GT41 gene family of GT-B glycosyltransferase superfamily [90]. Therefore, the carboxyl-terminal catalytic region in OGT has high homology, structural characteristics and catalytic activity of GT-B glycosyltransferases superfamily. Consistent with the structural characteristics of GT-B, OGT contains two similar Rossman folds separated by a deep fissure, which are called CDⅠ and CD Ⅱ, respectively, forming the catalytic core of OGT without the assistance of divalent metal ions [91]. CD Ⅰ mainly consists of a UDP identification pocket and some catalytic groups composed of acidic amino acids that stabilize the pyrophosphate bond through the synergistic action of divalent cations [92]. CD Ⅱ is a lectin-like domain that can be used to recognize and bind the glycoside of UDP-GlcNAc due to its strong affinity for carbohydrates caused by its own abundant trimer structure [93]. However, an Int-D exists in OGT and packs against the carboxyl-terminal catalytic lobe, which is distinct from the structural characteristics of GT-B superfamily [94]. It is preliminarily inferred that Int-D may interact with negatively charged membranes or nucleic acids based on the structural characteristics that contain an unusually large number of surface-exposed basic residues [82]. Meanwhile, PPO is located at the carboxyl terminal of OGT and strongly interacts with PIP3, which promotes the recruitment of OGT to the membrane under insulin induction for catalyzing the dynamic *O*-GlcNAcylation of the insulin signaling pathway [95]. Targeting special localization of this interaction led to the alteration in the phosphorylation of pivotal insulin signal molecules and weakening of the insulin signal transduction [96]. This suggests the indispensable role of OGT in diabetic pathology. In addition, there is a central flexible linker domain composed of about 120 amino acids [97]. The linker domain seems to exist only in metazoans without high conservatism and its function is unknown, which is the root cause of the difficulty in crystallization of OGT in higher metazoans [98]. The activity and substrate recognition of OGT are also regulated by phosphorylation [99]. For example, OGT is phosphorylated by GSK3β on Ser^3/4^, increasing in its own activity [100]. IRS phosphorylates OGT on Tyr^976^ with a similar effect [7]. OGT is phosphorylated by AMPK on Thr^454^, which changes its substrate-binding targets and subcellular localization [101]. The mutation of the phosphorylation site of OGT on Thr^12^ and Ser^56^ significantly changed the substrate binding of more than 500 proteins [102]. OGT is phosphorylated by CHK1 on Ser^20^, which changes its stabilization and required for cytokinesis [103]. OGT is also phosphorylated by CaMKII on Ser^20^, which increases its activity [104]. In addition, OGT is also *O*-GlcNAcylated by itself in Ser^3/4^ and Thr^1045^, and their role is unknown [7]. There are multiple acetylation sites in OGT [105]. The presence of these acetylation sites in the catalytic domain suggests that they may modulate OGT activity [18]. The advanced structure of OGT and the sites modified by various PTMs are shown in Figure 3C.

*Oga* is highly conserved in eukaryotic species, especially in mammals, but absent in prokaryotes and yeast [106]. *Oga* is mapped to chromosome 10 (10q24.32) as a single gene copy [107]. It is selectively spliced to produce ncOGA and sOGA, which are different at different carboxyl terminals [87]. Gene and protein structures of OGA are shown in Figure 3D,E. Cell fractionation analysis showed that ncOGA was mainly located in the cytoplasm, while the sOGA subtype existed in the nucleus [108]. ncOGA contains the amino-terminal catalytic domain and the central stalk domain and the carboxyl-terminal pseudo-HAT domain linked through two highly disordered (or low complexity) regions [109]. The amino-terminal catalytic domain of OGA is the GlcNAc hydrolysis domain with sequence homology to GH84 [110]. The stalk domain is a hinged region containing multiple alpha helices [111]. It is not conserved between species, which makes it a flexible region that facilitates the folding of the entire protein [112]. Although it has been reported that the HAT-like domain of ncOGA in mice has histone acetyltransferase activity in vitro, it has not been supported by more studies in vivo for lacking the critical residues for the binding of acetyl-coenzyme A [113]. However, the HAT-like domain is evolutionarily conserved, indicating that the pseudo-HAT domain may play an important role in the deglycosylation-associated functions [114]. sOGA lacks the HAT-like domain but contains 15 unique amino acid residues at the carboxyl terminal [115]. Interestingly, it has been reported that sOGA has higher hydrolytic activity in vitro. OGA preferentially removes GlcNAc from some sites, indicating that it has an equal cooperative relationship with OGT in regulating the replacement of *O*-GlcNAcylation [116]. The active form of OGA appears as homodimer [117]. OGA forms a homodimer in the form of arm to arm, in which the glycoside hydrolase domain of each monomer is covered by the stalk domain of another monomer, thus forming a potential substrate-binding cleft comprising conserved hydrophobic residues [97]. The glycopeptide of the *O*-GlcNAcylated protein is tightly bound in the substrate-binding cleft through the abundant GlcNAc contacts of the catalytic pocket in OGA, which involves the peptide side chain and the backbone interactions with cleft surface residues [118]. Meanwhile, OGA recognizes the specific characteristics of substrate peptides and hydrolyzes GlcNAc from a wide range of peptide sequences [119]. In addition, some specific residues on OGA contribute to its interaction with different peptide substrates, which means the differential regulation of *O*-GlcNAcylation on various proteins [120]. OGA is also affected by PTMs such as phosphorylation and *O*-GlcNAcylation [121]. There are abundant phosphorylation and ubiquitination sites in the domains of glycoside hydrolase and the HAT-like domain [7], but the effect of these modifications at corresponding sites on OGA activity remains to be further determined. The advanced structure of OGA and the sites modified by various PTMs are shown in Figure 3F. The *O*-GlcNAcylation of OGA at Ser^405^ is located in the central highly disordered region, suggesting a role in the regulation of OGA-OGT interactions because this is the binding region of OGA-OGT [122]. OGA is also SUMOylated at Lys^358^ and acetylated at Lys^599^, respectively [7,123].

### 2.2. Nutrient Availability Drives Global O-GlcNAcylation through HBP

**Figure 4:**
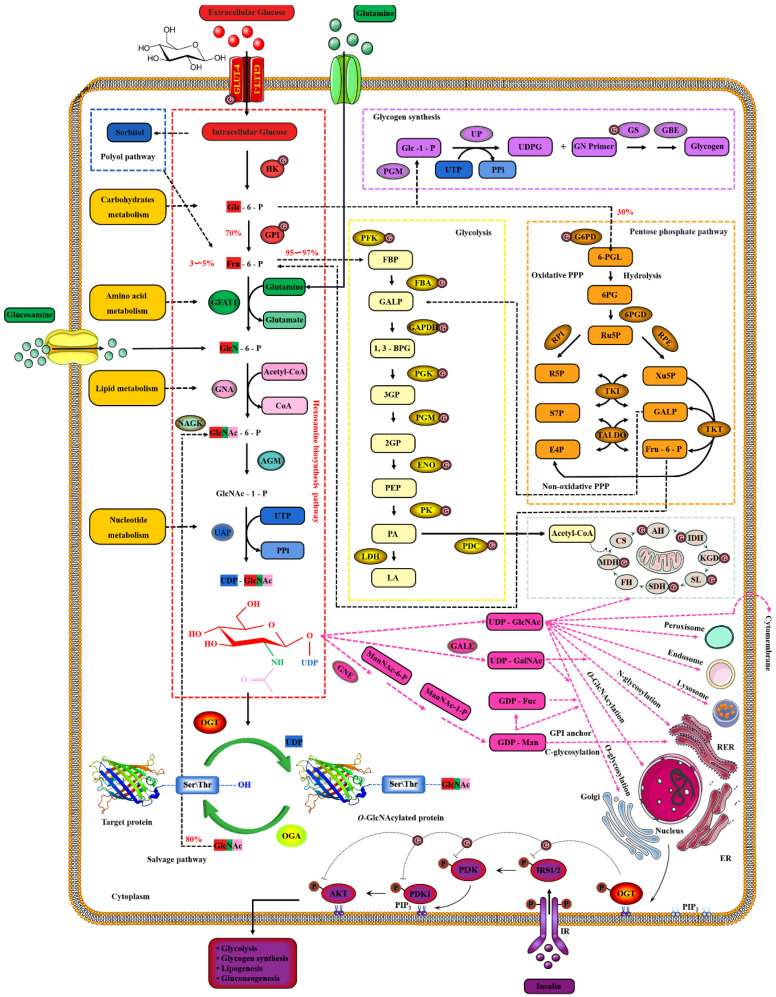
Nutrient availability drives the dynamic cycle of protein *O*-GlcNAcylation via the hexosamine biosynthesis pathway. The GlcNAc provided by UDP-GlcNAc is necessary and unique for *O*-GlcNAcylation, and the only source of UDP-GlcNAc is HBP. Changes in nutritional availability, such as carbohydrates, lipids, amino acids, nucleotides and ATP, fluctuate HBP flux. Therefore, UDP-GlcNAc and HBP link carbohydrate metabolism, lipid metabolism, amino acid metabolism and nucleotide metabolism. These physiological characteristics make OGT extremely sensitive to nutrient fluctuations.

Extracellular glucose is transported into the intracellular via GLUT-4 [124]. Only 2~3% of the intracellular glucose enters the HBP, while most of the remaining intracellular glucose enters the glycolysis, pentose phosphate pathway (PPP), glycogen synthesis and even polyol pathways, respectively [125]. Therefore, the *O*-GlcNAcylation cycle is strictly controlled by the flow of glucose through the HBP [126]. Initially, in a study, intracellular glucose was phosphorylated to Glc-6-P by HK, and then Glc-6-P was further isomerized to Fru-6-P by GPI [127]. Subsequently, 3~5% of Fru-6-P was added with an amino group from glutamine to synthesize GlcN-6-P and glutamate by GFAT, while the other 95% of Fru-6-P was used for glycolysis [128]. The enzymatic reaction is the rate-limiting step of HBP, and GFAT is also the key rate-limiting enzyme of HBP [129]. The activity of GFAT is still regulated by multiple pathways [130]. Firstly, the activity of GFAT is regulated by substrate availability, which is positively activated by the concentration of glucose and glutamine, and the negative feedback is inhibited by the concentration of UDP-GlcNAc and GlcN-6-P [131]. The activity of GFAT is also closely related to some PTMs. The Ser^243^ of GFAT is phosphorylated and its activity is reduced by AMPK, mTORC2 and CaMKII, and a similar effect is also caused by 2-Deoxy-D-glucose [132,133]. PKA also promotes the phosphorylation of GFAT at Ser^205/235^ [134]. Succinylation of GFAT at Lys^529^, acetylation of GFAT at Lys^114, 547, 650^ and multiple ubiquitination Lys sites of GFAT are predicted by PhosphoSitePlus^®^ v6.6.0.2 (https://www.phosphosite.org, accessed on 10 March 2022). Meanwhile, it has been reported that specificity protein 1, activating transcription factor 4 and X-box-binding protein 1 regulate GFAT at the transcriptional level [135,136]. Glutamine is necessary for this enzymatic reaction, but this restriction can be bypassed by glucosamine as an extended supplement [137]. Therefore, incubating cells with glucosamine or high concentration glucose or glutamine can bypass the rate-limiting step catalyzed by GFAT, thereby increasing global *O*-GlcNAcylation. GNA converts GlcNAc-6-P using acetyl-CoA [138]. Then, GlcNAc-6-P is catalytically translocated to GlcNAc-1-P by AGM [139]. It is worth noting that the only difference of HBP in prokaryotes is that GlcN-6-P is isomerized to GlcN-1-P and then GlcN-1-P is acetylated to form GlcNAc-1-P [140]. The HBP process in eukaryotes is as shown above. Finally, UTP is then utilized by UAP to convert GlcNAc-1-P into UDP-GlcNAc and release iPPi [141]. The HBP process involves the participation of glucose, glutamine, uridine, acetyl-CoA and ATP [29]. Therefore, UDP-GlcNAc, as the end-product of HBP, integrates the metabolisms of carbohydrates, amino acids, fats and nucleotides [142]. UDP-GlcNAc is a unique donor of *O*-GlcNAcylation, which provides GlcNAc, which is necessary and irreplaceable for *O*-GlcNAcylation [143]. GlcNAc provided by UDP-GlcNAc is used and transferred by OGT to the oxygen atom of the hydroxyl group of serine or threonine residues of the target protein [54]. On the contrary, the GlcNAc moiety is removed from *O*-GlcNAcylated proteins by OGA [144]. These hydrolyzed GlcNAc or other free GlcNAc obtained by lysosomal or nutrient degradation are converted to GlcNAc-6-P through N-Acetylglucosamine kinase (NAGK) and then used again for the synthesis of UDP-GlcNAc [145]. Therefore, GlcNAc can also bypass the rate-limiting step of HBP and GFAT, which is also effective for salvage pathways such as glucosamine and glutamine [146]. In addition, UDP-GlcNAc is also used as a substrate for the synthesis of proteoglycans, hyaluronic acid, glycolipids, GPI anchor, *N*-glycosylation and other *O*-glycosylation [147]. The activated UDP-GlcNAc is utilized by concentration-sensitive enzymes in the nucleus, cytoplasm and membrane to glycosylate the substrate or generate glucose conjugates [147]. UDP-GlcNAc is actively transported by nucleotide sugar transporters to cellular organelles, such as the ER and Golgi apparatus [148]. The differences in UDP-GlcNA permeability and relative cell volume of these organelles complicate the estimation of the cytoplasmic and nuclear concentrations of UDP-GlcNAc [149]. The relative abundance of *O*-GlcNAcylation is roughly negatively correlated with the more complex glycans [150]. These characteristics make UDP-GlcNAc and its derivatives extremely sensitive to the variations in cellular nutrients, so that the dynamic *O*-GlcNAcylaion can be used as a reporter of the functional status of multiple pathways and regarded as a metabolic sensor [24]. Meanwhile, the mutual conversion and complex relationship of the intermediate products in the HBP, polyol pathway, PPP, glycogen, glycolysis and TCA cycle intermediates greatly enlarge the nutritional sensitivity of *O*-GlcNAcylation [151] and also suggest the potential mechanism of *O*-GlcNAcylaion’s negative feedback regulation of these glucose metabolism branches. Indeed, *O*-GlcNAcylation is involved in multiple modes of metabolic regulation. Almost all the enzymes involved in glycolysis were identified to have been modified by *O*-GlcNAcylation [152]. The *O*-GlcNAcylated enzymes exist in every step of glycolysis, including GLUT4, HK, GPI, PFK, FBA, GAPDH, PGK, PDM, ENO, PK and PDC [59,153,154,155]. Glycogen synthesis is also regulated by *O*-GlcNAcylated GSK3β, and PPP activity is affected by *O*-GlcNAcylated G6PD [156,157]. In addition, increased HBP flux and *O*-GlcNAcylation also promotes fatty acid oxidation in the heart and adipose tissue [158]. The *O*-GlcNAylation of several transcription factors, such as PGC1α, FoxO3, NF-κB and CREB, also indirectly participates in transcriptional regulation of metabolism [159,160,161]. Although only briefly shown in Figure 4, it is worth noting that almost all enzymes in the TCA cycle are also modified by *O*-GlcNAcylation, such as AH, IDH, KGD, SL, SDH, MDH and the several subunits of respiratory chain complexes [162,163]. CS and FH may be potentially *O*-GlcNAcylatied, but there is still a lack of supporting evidence [152].

## 3. *O*-GlcNAcylation, Energy Metabolism and Insulin Sensitivity in Skeletal Muscle

Skeletal muscle is a repository of nutrients, enabling it to serve as the consumer and producer of energy during exercise, stress and starvation [164]. The energy requirements of skeletal muscle are enormous, and its energy expenditure increases 300 times from the base state to the full contraction state [165,166]. This directly affects glucose homeostasis in skeletal muscle, where CaMKII or GLUT4 is activated to increase glucose uptake [167]. In fact, skeletal muscle processes more than 80% of insulin to stimulate glucose uptake and is considered as one of the most critical insulin-sensitive tissues [168]. The metabolic flexibility in skeletal muscle ensures an adequate supply of energy for its work [169]. *O*-GlcNAcylation as a cellular nutrient sensor plays a key role in glucose metabolism during this physiological process [170]. In addition, skeletal muscle is the main target organ of insulin, and nutrient-driven *O*-GlcNAcylation is a key regulator of insulin signaling in skeletal muscle [62]. *O*-GlcNAcylation is considered to be critical in the dysregulation of the insulin signaling cascade and the molecular mechanism of insulin resistance [171].

### 3.1. O-GlcNAcylation Is the Key Regulator of Glucose Metabolism in Skeletal Muscle

Glucose uptake and disposal are the most important limiting factors in fuel metabolism and energy homeostasis of skeletal muscle [172]. Ample data show that *O*-GlcNAcylation regulates early glucose metabolism [159]. GLUT4 and its transport vesicle proteins in skeletal muscle and adipose tissue are modified and directly regulated by *O*-GlcNAcylation [173]. *O*-GlcNAcylation plays an essential role in maintaining glucose uptake by altering GLUT4 translocation, blocking GLUT4 phosphorylation, regulating GLUT4 downstream signal transduction or directly regulating vesicle proteins [174]. Hypoxia inducible factor 1α, a key transcriptional regulator of GLUT1, is modified by *O*-GlcNAcylation, which indirectly regulates and increases GLUT1 transcription and glucose uptake [175,176]. In addition, HK is a major regulator of cellular glucose uptake, and *O*-GlcNAcylated hexokinase IV or glucokinase in vivo/vitro positively regulates its expression, which is of positive significance in regulating glucose flux [177]. Similar changes have occurred in GPI [178]. The increase of global *O*-GlcNAcylation may further promote glucose into HBP and UDP-GlcNAc synthesis through these ways. The contribution of *O*-GlcNAcylation to glucose uptake reverses metabolic disorder, stress and cell death in skeletal muscle [27]. The increase of glucose uptake has been related to the increase of glycolytic flux and enzyme activity. It is important to note that almost all enzymes involved in the glycolysis pathway are *O*-GlcNAcylated, which modulates their expression or activity to participate in the regulation of glycolysis in skeletal muscle [152]. The *O*-GlcNAcylation of PFK at the Ser^529^ inhibits its activity and oligomerization, which redirects glucose flux into the PPP and reduces glycolytic flux [155]. PGK is a critical metabolic enzyme and catalyzes the production of the first ATP in the glycolysis pathway [179]. PGK is *O*-GlcNAcylated at the site of Thr^255^ [153]. The *O*-GlcNAcylation of PGK enhances its activity to promote glycolysis and translocation to mitochondria to inhibit the TCA cycle [180]. PFKFB3 is *O*-GlcNAcylated at Ser172 and competes with phosphorylation under metabolic stress [181]. PK is one of the main rate-limiting enzymes in glycolysis, and one of its subtypes is PKM2 [182]. The *O*-GlcNAcylation of PKM2 occurs at Thr^405^ and Ser^406^, which destroys its testamer stability, reduces its activity and causes its nuclear translocation [154,183]. These changes lead to the Warburg effect characterized by increased glucose consumption and lactate production [184]. The product pyruvate enters the anaerobic or aerobic pathway. The downstream LDH and PDC are also modified by *O*-GlcNAcylation [185]. These data suggest that *O*-GlcNAcylation plays a key regulatory role in the utilization of the glycolysis end-product. Note that almost all metabolic enzymes of the mitochondrial TCA cycle are also *O*-GlcNAcylated [186]. Meanwhile, *O*-GlcNAcylation modifies various mitochondrial proteins to change the morphology, function and quality of mitochondria [187,188]. For example, *O*-GlcNAcylation of PGC1ɑ is beneficial for maintaining mitochondrial biogenesis and metabolic reprogramming [189]. However, the mechanism of *O*-GlcNAcylation regulation on mitochondria affecting energy metabolism homeostasis and muscle fiber type switching in skeletal muscle is extremely complex, so it will not be discussed in detail here. Not all of these data are from skeletal muscle tissues or cell lines, and the exact role of *O*-GlcNAcylation in the regulation of energy metabolism in skeletal muscle remains to be further clarified [190]. Indeed, there is a complex relationship between myofibrils and the metabolic enzymes not limited to what we discussed above. For example, FBA is localized to the Z-line in association with α-actinin within the metabolon [191]. The interaction between FBA and downstream metabolic enzymes of glycolysis occurs in the thin filaments in the same pattern [192]. These specific interactions between glycolytic metabolon and the contractile apparatus may ensure a very efficient and dynamic localized production of ATP for myosin ATPase and actomyosin interactions resulting in force development [193]. In addition, the latest research found that the fluctuation of the global *O*-GlcNAcylation level also leads to the regulation of protein–protein interactions in multiple protein complexes [194]. Glycogen decomposition is conducive to maintaining the level of global *O*-GlcNAcylation. GSK3β is modified by *O*-GlcNAcylated, which may compete with the phosphorylation at Ser^9^ to inhibit its activity. The PPi required to produce UDPG is also *O*-GlcNAcylated, and the specific mechanism needs to be further confirmed [195]. These results suggest that *O*-GlcNAcylation is a regulator of glycogen synthesis. G6PD catalyzes the first speed-limiting step of PPP and is considered as the pacesetter of PPP and the main regulation point of NADPH production [196]. *O*-GlcNAcylation of G6PD at the site of Ser^84^ activates its activity and diverts glucose flow to PPP [197]. The absence of *O*-GlcNAcylation increases the secretion of IL-15 in skeletal muscle, which serves as a myokines regulating systemic oxidative metabolism [198]. Similarly, the secretion of myogenic IL-6 is indirectly regulated by *O*-GlcNAcylated p65 to maintain energy homeostasis in skeletal muscle [199]. In addition, creatine kinase is *O*-GlcNAcylated [200]. Creatine shuttle transmits information between ATP site consumption and mitochondria, so *O*-GlcNAcylation regulates this process.

### 3.2. O-GlcNAcylation-Mediated Insulin Sensitivity in Skeletal Muscle

HBP has long been known to be involved in glucose-induced insulin resistance. It has been widely recognized that the chronic high flow of HBP represents one of the mechanisms of insulin resistance caused by hyperglycemia [201]. Mice overexpressing GLUT1 in skeletal muscle have insulin resistance and are accompanied by medium- and long-term increased glucose flow and increased UDP-GlcNAc concentration in muscle [202]. Continuous exposure to high glucose or glucosamine can lead to impaired insulin stimulated GLUT4 translocation, resulting in subsequent reduced insulin-stimulated glucose uptake in muscle cells [168]. High glucose or glucosamine leads to the increase of HBP flux and UDP-GlcNAc level, in which glucosamine is more effective [203]. It is worth noting that these changes are related to the level of UDP-GlcNAc in the GLUT4-containing vesicles of the skeletal muscle under the condition of diabetes [204]. These data indicate that there is a strong correlation between HBP flux and UDP-GlcNAc level in skeletal muscle and insulin resistance and diabetes pathology. The catalytic activity of OGT is highly sensitive to HBP flux and UDP-GlcNAc concentration [6]. Therefore, elevated global *O*-GlcNAcylation levels in skeletal muscle induce insulin resistance [205]. Increasing UDP-GlcNAc enhanced many *O*-GlcNAcylated muscle proteins on bone through the co-infusion of insulin and glucosamine in a study [206]. Therefore, another possible mechanism of HBP induced insulin resistance is that the increase of *O*-GlcNAcylation of insulin signal-related proteins antagonizes their phosphorylation. PIP3 recruits OGT to the cytomembrane through the strong interaction with the PPO domain of OGT under diabetes or another insulin-insensitive state [95]. Meanwhile, OGT is phosphorylated at certain tyrosine residues and enhances its activity through the insulin-stimulated insulin receptor [207]. Subsequently, OGT catalyzes the dynamic *O*-GlcNAcylation of IRS, PDK1, AKT, FoxO1 and other insulin signal molecules [51]. The *O*-GlcNAcylation of IRS-1 at Ser^1101^ and IRS-2 at Ser^1149^ inhibits their phosphorylation at the same site, resulting in the attenuation of the insulin signal [208]. The *O*-GlcNAcylation of AKT at the Thr^305^ and Thr^312^ inhibits its phosphorylation atThr^308^ through disrupting the interaction between AKT and PDK1 [209,210]. Therefore, excessive nutrients, such as glucose and fatty acids, lead to an abnormal increase in global *O*-GlcNAcylation, which reduces insulin signal transduction efficiency, produces insulin resistance and forms a vicious circle and glucose toxicity [211].

## 4. *O*-GlcNAcylation Is an Emerging Mediator of Contractile and Structural Properties in Skeletal Muscle

### 4.1. O-GlcNAcylation Is an Essential Regulator of Contractile Properties in Skeletal Muscle

To date, many *O*-GlcNAcylated contractile proteins and contractile-related regulatory proteins in sarcomere have been identified [67]. These key contractile proteins include actin, myosin, MLC and MHC, tropomyosin and troponin, etc. [55,66,212]. In view of this, the physiological role of *O*-GlcNAcylation on skeletal muscle contractile activity has been concerned. Many data emphasize that *O*-GlcNAcylation mediates calcium activation properties to regulate the contractile activity of skeletal muscle. Increased *O*-GlcNAcylation of MHC, α-tropomyosin and α-sarcomeric actin in myocardium of diabetic mice resulted in the decrease of sarcomere calcium sensitivity [213]. Reversible reductions in calcium affinity and sensitivity of muscular fibers occur when exposed to GlcNAc, and *O*-GlcNAcylation of some critical contractile proteins increased, such as MHC, MLC and actin [214]. Moreover, similar results appeared in skinned fibers and cardiac trabeculae. Phosphorylation of Tn I at the Ser^23^ and Ser^24^ by PKA improves the calcium sensitivity of cardiomyocytes and alter of myofilament properties [215]. Interestingly, Tn I, Tn T and Tn C of the troponin complex are *O*-GlcNAcylated in skeletal muscle and myocardial tissue [216]. The *O*-GlcNAcylation levels of Tn I in fiber cells exposed to GlcNAc or OGA inhibitors increased, and the calcium activation properties reduced [217]. However, this treatment did not affect the phosphorylation levels of Tn I at Ser^23/24^ [218]. One of the possible mechanisms is that the GlcNAc part destroys the protein–protein interaction [212]. For example, weak electrostatic force maintains the interaction between tropomyosin and actin and *O*-GlcNAcylation just change specific electrostatic charges [219]. Four *O*-GlcNAcylated sites were found on myosin in skeletal muscle. These sites are located in the marginal of the carboxy terminal, which is closely related to the polymerization and the interaction of MHC [220]. Further evidence is that one of the sites is associated with a hereditary myosin myopathy because its mutation destroys the polymerization of myosin to myomesin, M protein and titin [67]. Unfortunately, although these specific *O*-GlcNAcylation changes are associated with calcium sensitivity, they have not been appreciated and studied, and precise *O*-GlcNAcylated site identification is lacking.

Many reports have shown that *O*-GlcNAcylation may play an equally crucial role in skeletal muscle physiology as phosphorylation [65,67,221,222]. Another possible mechanism is that *O*-GlcNAcylation plays its biological function by interacting with phosphorylation, as shown in Box 1. For example, the potential antagonism between the *O*-GlcNAcylation at Ser^190^ and the phosphorylation at Ser^208^ of Tn T plays a therapeutic role in the course of ischemic heart failure [223]. It must be recognized that MLC2 is the most suitable example to explain this mechanism. The elevated sarcoplasmic Ca^2+^ is bound to four divalent metal-binding sites on calmodulin to form the “Ca_4_^2+^—calmodulin” complex [224]. The complex then interacts with the inactive catalytic subunit of myosin light chain kinase (MLCK) to form an active holoenzyme complex, namely “Ca_4_^2+^—calmodulinMLCK” [225]. The “Ca_4_^2+^—calmodulin—MLCK” complex phosphorylates the slow fiber subtype MLC2 at the Ser^14^ [226]. Meanwhile, MLC2 non-covalently surrounds the neck region of myosin and provides it with additional and powerful mechanical support [227]. This process is Ca^2+^/calmodulin dependent, and the fast fiber subtype MLC2 is phosphorylated at Ser^15^ [228]. This effect is eliminated by the recognition and dephosphorylation of MLC2 by MYPT and PP1 [229,230]. The phosphorylation of MLC2 is an pivotal regulation to enhance the contractile capacity of sarcomeres by increasing calcium sensitivity, although it is not necessary for contraction [231]. These data also show that MLC2 phosphorylation causes changes in the structure of thick filaments and increase the number of cross bridges and their attachments because calcium sensitivity is directly proportional to the ATPase activity of actomyosin [232]. It is further explained that the electrostatic repulsion between the negative charge of phosphate added on MLC and MHC causes the position of myosin head to move, thus promoting the formation of cross bridges [233]. MLC2 phosphorylation is slower than contraction in terms of kinetics, which is regarded as a biochemical memory [234]. This helps to fight muscle fatigue by enhancing muscle mechanical function during prolonged or repeated activities [231]. MLC2 was found to be *O*-GlcNAcylated in the myocardium and skeletal muscle. *O*-GlcNAcyation of MLC2 in myocardial tissue occurred at the site of Ser^15^. It is worth noting that the only phosphorylation site overlaps with the only *O*-GlcNAcyation site on MLC2. This suggests that the close potential interaction between *O*-GlcNAcyation and phosphorylation of MLC2 is in the calcium activation properties of sarcomere. This interaction has been proved to be mutually exclusive, and the dynamics of this interaction vary according to the pattern of skeletal muscle activity [235]. The precise regulation of phosphorylation and *O*-GlcNAcyation on MLC2 involves a multienzyme cluster. This multienzyme cluster contains MLCK/MYPT2/PP1 and OGT/OGA, which are involved in the phosphorylation and *O*-GlcNAcyation of MLC2 [226]. This multienzyme cluster is preferentially located at the Z disk of the sarcomere and responds to the physiological signals of skeletal muscle to strengthen the interaction between the enzymes contained in itself [236]. For example, the partial recombination of this multienzyme clusters in skeletal muscle dysfunction, such as increased co-localization between MLCK and OGA. The location of OGT and OGA in the diabetic heart is redistributed in the sarcomere [237]. This is due to enhanced OGA activity and increased the interaction with α-actin, tropomyosin and MLC1, while OGT was the opposite. This results in the removal of abnormal *O*-GlcNAcylation to restore myofilaments to Ca^2+^ response [238]. Nevertheless, the exact effect and mechanism of *O*-GlcNAcylation on MLC2 need to be further confirmed. One credible assumption is that *O*-GlcNAcylation causes steric hindration between MLC2 and MHC due to its stokes radius being many times larger than phosphate [19].

In general, *O*-GlcNAcylation is considered as a new mechanism to regulate the contractile properties of skeletal muscle by modifying critical contractile proteins to regulate the interaction between other proteins or with itself, and phosphorylation to mediate calcium activation. In addition, the regulatory effect of *O*-GlcNAcylation on the contractile activity of skeletal muscle is also related to its effect on sarcomere structure [239], which isdiscussed in the next section.

### 4.2. O-GlcNAcylation Is an Emerging Maintainer of the Structural Properties in Skeletal Muscle

**Figure 5:**
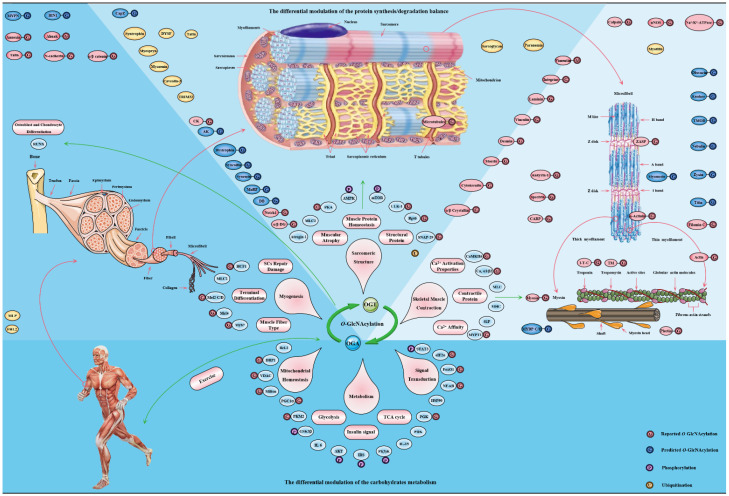
The major targets and pathways shown to be altered by *O*-GlcNAcylation in in the skeletal muscle physiology. Thousands of *O*-GlcNAcylated proteins have been identified in skeletal muscle cells. These *O*-GlcNAcylated proteins are classified into contractile proteins, sarcolemma proteins, structural proteins and cytoskeletal proteins, as well as mitochondrial proteins, metabolic enzymes, transcription factors and signal proteins. Therefore, the effects of *O*-GlcNAcylation on various physiological processes of skeletal muscle may be realized from the following four aspects: (**1**) its regulation of carbohydrate metabolism with sensing nutritional availability; (**2**) its maintenance of structural protein synthesis/degradation balance; (**3**) its improvement of sarcomere contractile activity by modulating the calcium activation properties; (**4**) its promotion of adaptation and protection under exercise and certain adverse circumstances.

Skeletal muscle is an extremely complex and organized machine with a unique stripe morphological characteristic [240]. This is based on the precise assembly and regular arrangement of sarcomeres with the combination of myofibrillar proteins and structural proteins [241]. Not only the actin and myosin discussed above, but also many critical myofibrin proteins involved in sarcomere structure are modified by *O*-GlcNAcylation, including as actinin, desmin, titin, ZASP, filamin C, myomesin, myopalladin, plectin, BAG3, etc. [217,242]. Meanwhile, a variety of pivotal structural proteins form a complex and ordered “sarcomere cytoskeleton”, and their interactions are essential for maintaining the basic structure of sarcomere and performing physiological functions of sarcomere such as contraction [243]. Moreover, the nodes of multiple dynamic interactions between these various proteins are in the Z disk, M line and I band [244]. Most of the above pivotal structural proteins involved in sarcomere structure are modified by *O*-GlcNAcylation, including as cytokeratin, laminin, spectrin, α/β-crystallin, integrin, vinculin, etc. [245,246,247,248]. First of all, the increased overall O-GlcNAcylation level in C2C12 myotubes resulted in an increase in the width of A band and M line in a study, while the width of I band and sarcomere length decreased [247]. These changes in the morphometric parameters of sarcomere caused by the fluctuation of *O*-GlcNAcylation level are the most powerful evidence to support that *O*-GlcNAcylation is an essential modulator of sarcomere structure. Phosphorylation is known to significantly regulate protein–protein interactions on sarcomeres, such as titin-myomesin and ZASP-myotilin interactions [249]. Hence, the dynamic balance between O-GlcNAcylation and phosphorylation may affect sarcomere structure. For example, the Z disk is the most concerned node of both O-GlcNAcylation and phosphorylation [249]. Desmin is capable of multiple PTMs, such as O-GlcNAcylation, phosphorylation and ubiquitination. It is recognized that phosphorylation regulates the polymerization of desmin [250]. *O*-GlcNAcylation also regulates the polymerization of desmin, just as it regulates the polymerization of tubulin and cytokeratin filaments 8/18 [251,252,253]. α/β-crystallin serve as molecular chaperones to facilitate the localization, aggregation, and assembly of desmin [254]. *O*-GlcNAcylation of α/β-crystallin at Thr^170^ and Thr^162^ regulates its localization and its interaction with desmin, respectively [255,256]. The multiple *O*-GlcNAcylation sites of mouse titin are located on the kelch-12 domain, and the absence of titin leads to the change of muscle structure and the decrease of muscle performance [249,257]. These *O*-GlcNAcylation sites are located in the key regions of sarcomere assembly and myosin polymerization and its interaction with MyBP-C and MHC [258,259]. Interestingly, *O*-GlcNAcylation sites of MHC have also been found. These sites are adjacent to its domain of polymerization and interaction with myosin and titin [220]. *O*-GlcNAcylation of MHC is located at Ser^1708^, and it is further considered to be involved in Laing early-onset distal myopathy due to its proximity to mutant Leu^1706^ residue. Meanwhile, *O*-GlcNAcylation sites in the PxxP domain of BAG3 and the plakin domain repeat B5 of plectin have been identified, which are related to the interaction with SH3-containing protein and intermediate filament proteins, respectively [249]. This evidence suggests that *O*-GlcNAcylation occurs in specific domains of certain structural proteins to regulate interactions other proteins for maintaining sarcomere structure and function. This view was confirmed by a recent study that *O*-GlcNAcylated milton binds to FHL2 to anchor mitochondria to F-actin [221,260]. The main targets and pathways of *O*-GlcNAcylation in skeletal muscle physiology are shown in Figure 5.

## 5. Conclusions and Perspectives

In the past few decades, the molecular mechanism and biological function of *O*-GlcNAcylation have been thoroughly studied. However, the physiological mechanisms by which OGT and OGA accurately identify thousands of substrates and dynamically maintain their *O*-GlcNAcylation homeostasis remain to be further clarified. One of the current obstacles is that it is difficult to obtain OGT and OGA crystals in higher organisms. Another obstacle is the identification of precise sites of *O*-GlcNAcylation. This will be an urgent difficulty to overcome and a research node worth exploring. Meanwhile, the research of efficient and specific inhibitors of OGT and OGA has always been a hotspot. The solution of these difficulties will depend on innovative biotechnology and strategies in the future. For example, the calculation and prediction of *O*-GlcNAcylation site of proteins is an interesting research direction.

*O*-GlcNAcylation-mediated glucose homeostasis and the insulin sensitivity of skeletal muscle endow the plasticity of metabolic properties to adapt to nutritional availability and physiological clues. However, changes in the fine characterization of global *O*-GlcNAcylation of skeletal muscle are extremely complex, depending on muscle fiber type, abandonment, rest and exercise patterns such as type and intensity. However, we have not discussed these factors in this review due to the limited space. Further study on the metabolic regulation difference of *O*-GlcNAcylation in basal and exercise states is helpful to explore and understand how *O*-GlcNAcylation responds to and coordinates various molecular signals.

The role of abnormal *O*-GlcNAcylation levels in the pathophysiology of various cancers, neurodegeneration, obesity, diabetes and its complications were also highlighted. However, these research studies have only explored the tip of the iceberg of the pathological role of *O*-GlcNAcylation, which needs to be studied further. There has been a lot of evidence to support the essential role of *O*-GlcNAcylation in skeletal muscle physiology and pathology, but these studies have been underestimated and ignored. The abnormality of global *O*-GlcNAcylation level is one of the pathogenic factors of skeletal muscle atrophy [226,261]. Destruction of OGA activity mediates high levels of *O*-GlcNAcylation, resulting in muscle atrophy [262]. One possible underlying mechanism is that *O*-GlcNAcylation appears to negatively regulate myogenesis. Increased global *O*-GlcNAcylation was shown to inhibit the terminal differentiation program of myogenesis in skeletal muscle [263]. The *O*-GlcNAcylation of Mef2D on its DNA-binding and transactivation domain, which is reduced by myogenic stimulation, inhibits its recruitment to the myogenin promoter [264]. Another possible mechanism is that *O*-GlcNAcylation involves the regulation of some catabolic and anabolic pathways, leading to atrophy, such as increased Murf-1 expression [261]. Proteostasis of all components of the cytoskeletal framework is necessary for the structure and function of skeletal muscle [265]. Due to autophagy and proteasome degradation pathways, skeletal muscle effectively recycles damaged or aged organelles and accumulated protein aggregates and breaks down proteins to meet the body’s energy needs [266]. With the continuous understanding of the extensive effects of *O*-GlcNAcylation on cell function, the benefits of *O*-GlcNAcylation in mediating autophagy, apoptosis and proteasome have been recognized [47]. In addition, the fine characterization changes of *O*-GlcNAcylation in muscle fibers in muscular dystrophy, myositis and rhabdomyolysis have been researched [64]. This adverse effect occurs in myocardium and smooth muscle, rather than being confined to skeletal muscle, such as cardiovascular diseases and vasculopathy caused by diabetes [70,267]. However, these potential pathogenic mechanisms are extremely complex, and we did not discuss them in this review due to limited space. Further research on the role of *O*-GlcNAcylation in the pathology of these skeletal muscle diseases will provide us with a variety of new therapeutic targets in the future. In particular, the further studies of *O*-GlcNAcylation mediated skeletal muscle autophagy and its physiological mechanism in muscle mass and atrophy and systemic energy metabolism will help to find emerging signaling pathways.

What is describe in this review is only a superficial and less in-depth understanding of the role of *O*-GlcNAcylation in skeletal muscle physiology. We believe that the exploration of *O*-GlcNAcylation-mediated differential regulation of skeletal muscle energy metabolism under various exercise modes and the discovery of the potential mechanism of *O*-GlcNAcylation in various skeletal muscle pathologies will be a vigorous research hotspot and direction in the future. Over time, these studies will provide new valuable insights in the fields for skeletal muscle diseases and exercise rehabilitation.

## Data Availability

Not applicable.

## Supplementary Materials

The following are available online at https://www.mdpi.com/article/10.3390/cells11111789/s1, Table S1: Types and process of protein glycosylation, Table S2: Common tools for studying, identification, and detection of O-GlcNAcylation.

## Abbreviations

1,3BPG	1,3-bisphosphoglycerate
2PG	2-phosphoglycerate
3PG	3-phosphoglycerate
5mC	5-methylcytosine
5S-GlcNAc	5-thio-N-acetylglucosamine
5S-GlcNHex	2-deoxy-2-N-hexanamide-5-thio-d-glucopyranoside
6PGD	6-phosphogluconate dehydrogenase
6-PGL	6-phosphogluconolactone
AGM	phosphoacetylglucosamine mutase
AH	aconitate hydratase
AK	adenylate kinase
AMPK	AMP-activated protein kinase
Ara	L-arabinose
BAG	benzyl-2-acetamido-2-deoxy-D-galactopyranoside or GalNAc-α-O-benzyl for short
BAG3	BCL2-related athanogene 3
BIN1	bridging integrator 1
BZX	4-methoxyphenyl 6-acetyl-2-oxo-2,3-dihydro-1,3-benzoxazole-3-carboxylate;
CaMKIIδ	calcium-calmodulin-dependent protein kinase 2 delta
CaMKIV	calcium-dependent protein kinase type IV
CARP	cardiac ankyrin repeat protein
Cav α1/β2	L-type Ca2+ channel subunit alpha 1 and beta 2
CBP	CREB-binding protein
CD I	catalytic domain I
CD II	catalytic domain II
CK	creatine kinase
COLGALT	collagen O-Gal transferase
CREB	cyclic AMP-response element-binding protein
CS	citrate synthase
DB	dystrobrevin
Dia	DiActrideoxyhexose
DOGT	GlcNAc transferase
Dol-P-Man	dolichol phosphate mannose
Dol-PP-Oligo	dolichylpyrophosphate Glc_3_Man_9_GlcNAc_2_
DON	6-Diazo-5-oxo-L-norleucine
DYSF	dysferlin
E4P	erythrose-4-phosphate
EGF	epidermal growth factor-like repeat
ENO	beta-enolase
ER	endoplasmic reticulum
EthNP	ethanolamine phosphate
EZH2	enhancer of zeste homolog 2
FBA	fructose-1,6-bisphosphate aldolase
FBP	fructose-1,6-bisphosphate
FH	fumarate
FHL2	four and a half LIM domains protein 2
Fru-6-P	fructose-6-phosphate
Fuc	L-fucose
FucNAc	N-Acetyl-fucosamine
G6PD	glucose-6-phosphate dehydrogenase
Gal	D-galactose
GALE	UDP-galactose-4-epimerase
GalNAc	N-acetyl-D-galactosamine
GALNT	polypeptide GalNAc transferase
GALP	glyceraldehyde-3-phosphate;
GAPDH	glyceraldehyde phosphate dehydrogenase
GBE	Glycogen-branching enzyme
GDP-Fuc	GDP-fucose
GDP-Man	GDP-Mannose
GFAT	glutamine Fru-6-P aminotransferase
GH84	glycoside hydrolase family 84
Glc	D-glucose;
Glc-6-P	glucose-6-phosphate
GlcN-6-P	glucosamine-6-phosphate
GlcNAc	*N*-acetylglucosamine
GlcNAc	*N*-acetyl-D-glucosamine;
GlcNAc-1-P	*N*-acetylglucosamine-1-phosphate
GlcNAc-6-P	*N*-acetylglucosamine-6-phosphate
GN	glycogenin
GNA	glucosamine-6-phosphate N-acetyltransferase 1
GNE	UDP-GlcNAc 2-epimerase/ManNAc kinase
GPI	Glc-6-P isomerase
GPI	glycosylphosphatidylinositol
GS	glycogen synthase
GYG	glycogenin
HAT	histone acetyltransferase
HBP	hexosamine biosynthesis pathway
HDAC4	histone deacetylase 4
HK	hexokinase
Hyl	hydroxylysine
IDH	isocitrate dehydrogenase
Int-D	intervention domain
I-T-C	troponin complex (Tn I, Tn T, Tn C)
KGD	ketoglutarate dehydrogenase
LA	lactate
LDH	lactate dehydrogenase
Man	Mannose
ManNAc	*N*-acetylmannosamine
MDH	malate dehydrogenase
Mef2 C/D	myocyte-specific enhancer factor 2 C/D
MHC	myosin heavy chain
MLC2	myosin light chain 2
MLP	muscle LIM protein
mOGT	mitochondrial OGT
MyBP-C	Myosin-binding protein C
MYPN	myopalladin
MYPT1	myosin phosphatase target subunit 1
NButGT	1,2-dideoxy-2’-propyl-alpha-D-glucopyranoso-[2,1-D]-Delta 2’-thiazoline
ncOGA	nucleocytoplasmic OGA
ncOGT	nucleocytoplasmic OGT
OGA	*O*-GlcNAcase
OGT	*O*-GlcNAc transferase
OST	oligosaccharyltransferase
PA	pyruvate
PDC	pyruvate dehydrogenase complex
PEP	phosphoenolpyruvate
PFK	phosphofructokinse
PGK	phosphoglycerate kinase
PGM	phosphoglucomutase
PIP3	phosphatidylinositol (3,4,5)—triphosphate
PK	pyruvate kinase
PKA	cAMP-dependent protein kinase
POFUT	protein O-fucosyltransferase
POGLUT	protein O-Glc transferase
POMT	protein O-Man transferase
PP1	protein phosphatase 1
PP2A	protein phosphatase 2α
PPi	inorganic phosphate
PPO	Phosphoinositide-binding domain
Pse	pseudaminic
PTase	phosphoglycosyltransferase
PTM	post-translational modification
PUGNAc	O-(2-acetamido-2-deoxy-D-glucopyranosylidene) amino N-phenyl carbamate
PY19L	dpy-19 like C-Man transferase
R5P	ribose-5-phosphate
RER	rough endoplasmic reticulum
Rha	L-rhamnose
RPE	Ru5P epimerase
RPI	Ru5P isomerase
Ru5P	ribulose-5-phosphate
S1P	sphingosine-1-phosphate
S7P	sedoheptulose-7-phosphate
SC	satellite cell
SDH	succinate dehydrogenase
SIN3A	SIN3transcription regulator family member A
SIRT1	sirtuin 1
SL	succinyl-CoA ligase
sOGA	short OGA
sOGT	short OGT
STZ	streptozotocin
TALDO	transaldolase
TET	ten-eleven translocation protein
Thiamet G	O-(2-acetamido-2-deoxy-D-glucopyranoseylidene)
TKT	transketolase
TM	tropomyosin
TMOD	tropomodulin
TPR	tetratripeptide repeat domain
TRIM32	tripartite motif protein
TT04	3-[2-(1-adamantyl)ethyl]-2-(4-chlorophenyl)imino-4-oxo-1,3-thiazinane-6-carboxylic acid
UAP	UDP-N-acetylglucosamine pyrophosphorylase
UDP-5S-GlcNAc	uridine diphospho-5-thio-N-acetylglucosamine or Ac4-5SGlcNAc
UDPG	UDP-Glucose
UDP-Gal	UDP-galactose
UDP-GalNAc	UDP-*N*-acetylgalactosamine
UDP-GlcNAc	UDP-*N*-acetylglucosamine
UDP-Xyl	UDP-xylose
UP	UDP-Glucose pyrophosphosphprylase
UTP	uridine triphosphate
Xu5P	xylulose-5-phosphate
Xyl	D-xylose
XYLT	protein *O*-Xyl transferase
ZASP	Z-band alternatively spliced PDZ-motif protein
α/β-DG	α/β-dystroglycan
α-GlcNAc Thiolsulfonate	2-acetamido-2-deoxy-1-S-(4-methylbenzenesulfonyl)-1-thio-α-D-glucopyranose
