# Supplementary material for: O-GlcNAcylation: The Underestimated Emerging Regulators of Skeletal Muscle Physiology
Source: Cells. 2022 May 30;11(11):1789. doi: 10.3390/cells11111789 (PMC9180116; doi:10.3390/cells11111789)
Supplement: Supplementary file 1 [file cells-11-01789-s001.zip › cells-1684182_supplementary.pptx]

## Slide 1
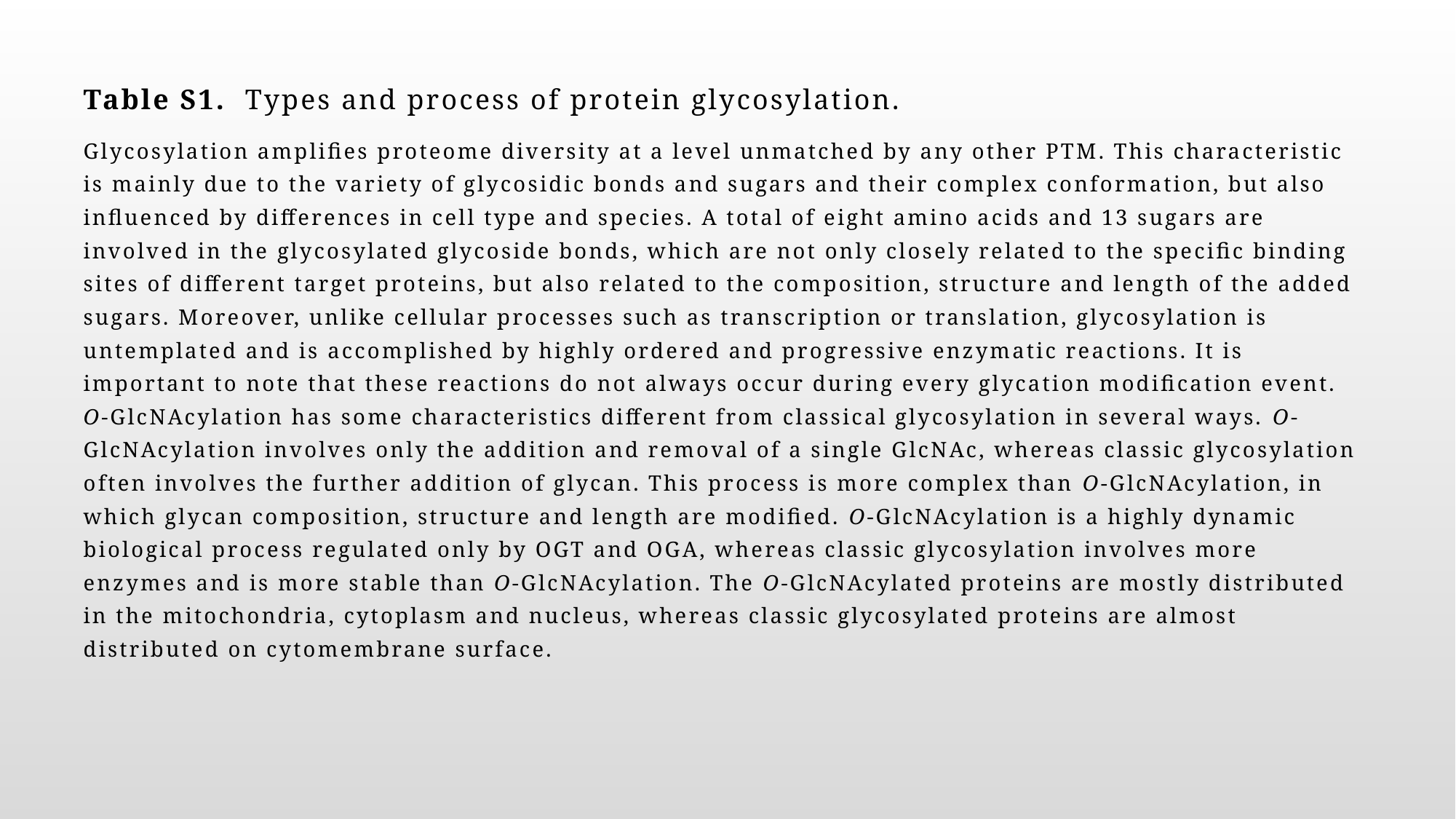

Table S1.  Types and process of protein glycosylation.
Glycosylation amplifies proteome diversity at a level unmatched by any other PTM. This characteristic is mainly due to the variety of glycosidic bonds and sugars and their complex conformation, but also influenced by differences in cell type and species. A total of eight amino acids and 13 sugars are involved in the glycosylated glycoside bonds, which are not only closely related to the specific binding sites of different target proteins, but also related to the composition, structure and length of the added sugars. Moreover, unlike cellular processes such as transcription or translation, glycosylation is untemplated and is accomplished by highly ordered and progressive enzymatic reactions. It is important to note that these reactions do not always occur during every glycation modification event. O-GlcNAcylation has some characteristics different from classical glycosylation in several ways. O-GlcNAcylation involves only the addition and removal of a single GlcNAc, whereas classic glycosylation often involves the further addition of glycan. This process is more complex than O-GlcNAcylation, in which glycan composition, structure and length are modified. O-GlcNAcylation is a highly dynamic biological process regulated only by OGT and OGA, whereas classic glycosylation involves more enzymes and is more stable than O-GlcNAcylation. The O-GlcNAcylated proteins are mostly distributed in the mitochondria, cytoplasm and nucleus, whereas classic glycosylated proteins are almost distributed on cytomembrane surface.

## Slide 2
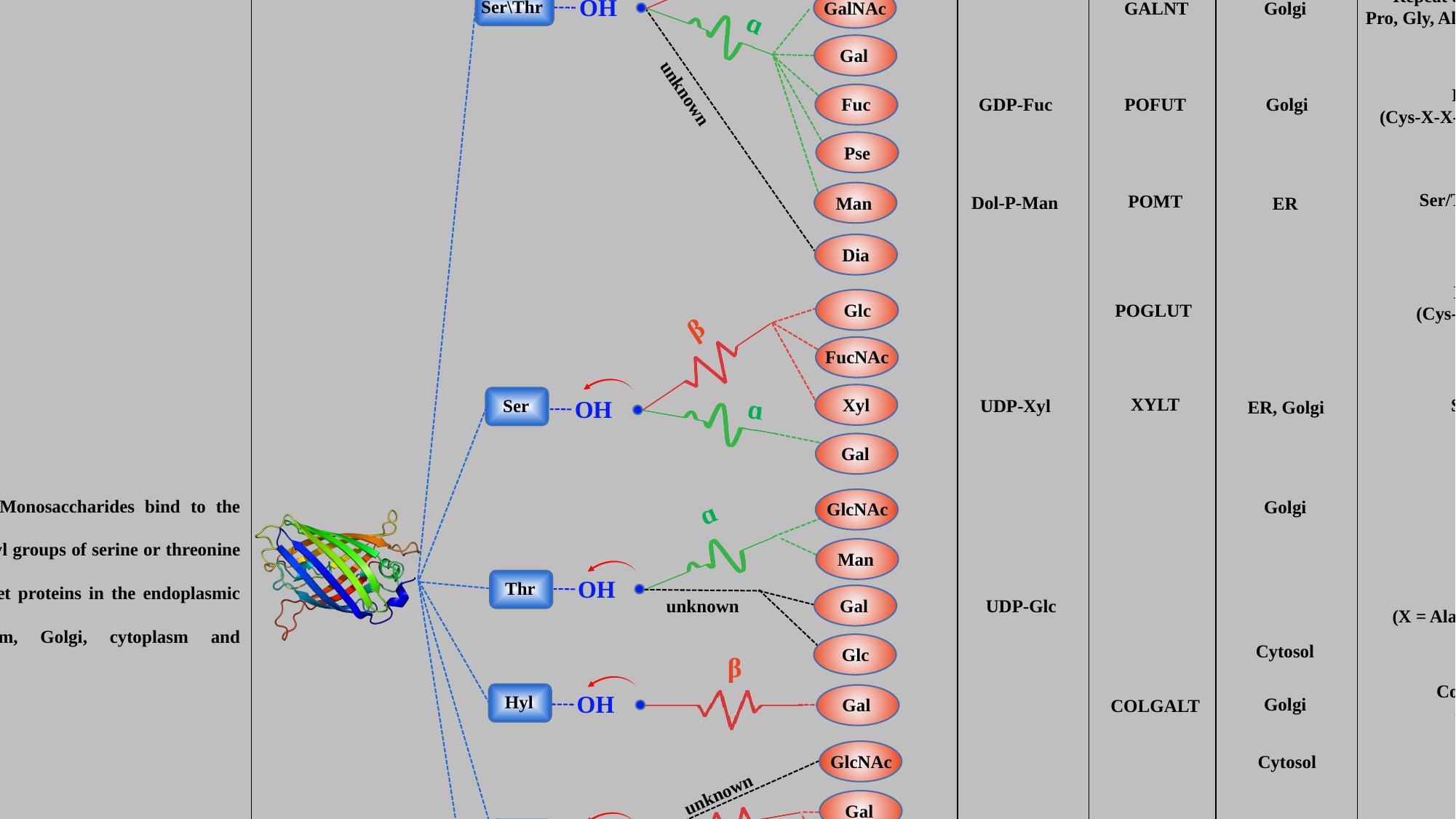

Phylogenetic distribution
Linkage diagram
Types
Location
Prokaryote
Enzyme
Glycosylation motifs
Description
Glycosyl donor
Eukaryote
Configuration
Sugar
Target protein
Amino acid residue
 Archaea
Bacteria
Yes
Yes
Yes
GlcNAc
Dol-PP-Oligo
ER
β
Glc
Yes
No
Yes
 Glycans bind to the amino-group of asparagine on the target protein in the rough endoplasmic reticulum
N2H
Asn
Asn-X-Ser/Thr
 (X = any amino acid except Pro)
GalNAc
No
No
Yes
unknown
OST
N-glycosylation
Rha
No
Yes
No
β
N2H
Arg
Glc
Asn-X-Ser/Thr
Yes
No
No
Ser/Thr rich domains
（near Pro, Val, Ala, Gly）
Yes
No
No
GlcNAc
Cytosol, nucleus
UDP-GlcNAc
OGT
β
No
No
Yes
GalNAc
UDP-GalNAc
 Repeat domains rich in Ser, Thr, Pro, Gly, Ala
OH
Yes
No
No
Ser\Thr
GalNAc
GALNT
Golgi
ɑ
Gal
Yes
Yes
No
unknown
EGF modules
 (Cys-X-X-Gly-Gly-Thr/Ser-Cys)
Fuc
GDP-Fuc
Golgi
POFUT
Yes
No
No
Pse
No
No
Yes
Man
Ser/Thr rich domains
POMT
Dol-P-Man
ER
Yes
No
No
Dia
No
No
Yes
EGF modules
 (Cys-X-Ser-X-Pro-Cys)
Yes
No
No
Glc
POGLUT
β
FucNAc
No
Yes
No
Xyl
ɑ
Yes
No
No
Ser
XYLT
Ser-Gly (Ala)
OH
UDP-Xyl
ER, Golgi
Gal
Yes
No
No
 Monosaccharides bind to the hydroxyl groups of serine or threonine on target proteins in the endoplasmic reticulum, Golgi, cytoplasm and nucleus
GlcNAc
Golgi
Yes
No
No
ɑ
Man
Yes
No
No
OH
Thr
O-glycosylation
Gly-X-Thr
(X = Ala, Arg, Pro, Hyp, Ser)
Gal
unknown
UDP-Glc
Yes
No
Yes
Glc
Cytosol
Yes
No
No
β
Collagen repeats
(X-Hyl-Gly)
OH
Hyl
Gal
Yes
No
No
Golgi
COLGALT
GlcNAc
Hyp143
Yes
Yes
No
Cytosol
unknown
Gal
Yes
No
No
OH
Hyp
β
Ara
Yes
No
No
ɑ
Repetitive Hyp rich domains
 (e.g., Lys-Pro-Hyp-Hyp-Val)
Ara
Yes
No
No
β
Glc
No
Yes
No
Gal
OH
Tyr
No
Yes
No
ɑ
Tyr194 in glycogenin
(autoglucosylation)
Glc
Yes
No
No
Cytosol
GYG
 Mannose binds to the indole ring of tryptophan on target protein in the endoplasmic reticulum
Trp
Man
ER
DPY19L
Trp-X-X-Trp
C-mannosylation
Yes
No
No
ɑ
 The glycan core connects the phospholipid to the c-terminal of the target protein in the endoplasmic reticulum
 GPI attached after cleavage of C-terminal peptide
Man
COOH
EthNP
Transamidase
ER
GPI
Yes
No
Yes
Glypiation
Ser rich domains
(e.g., Ala-Ser-Ser-Ala)
ɑ
Yes
No
No
GlcNAc
Golgi
P
P
P
P
1
1
1
1
 Glycans bind to the target protein serine in Golgi through phosphodiester bond
Ser rich repeat domains
Yes
No
No
Man
GDP-Man
β
Golgi
OH
Ser
PTase
Phosphoglycosylation
Fuc
Yes
No
No
unknown
Xyl
Yes
No
No

## Slide 3
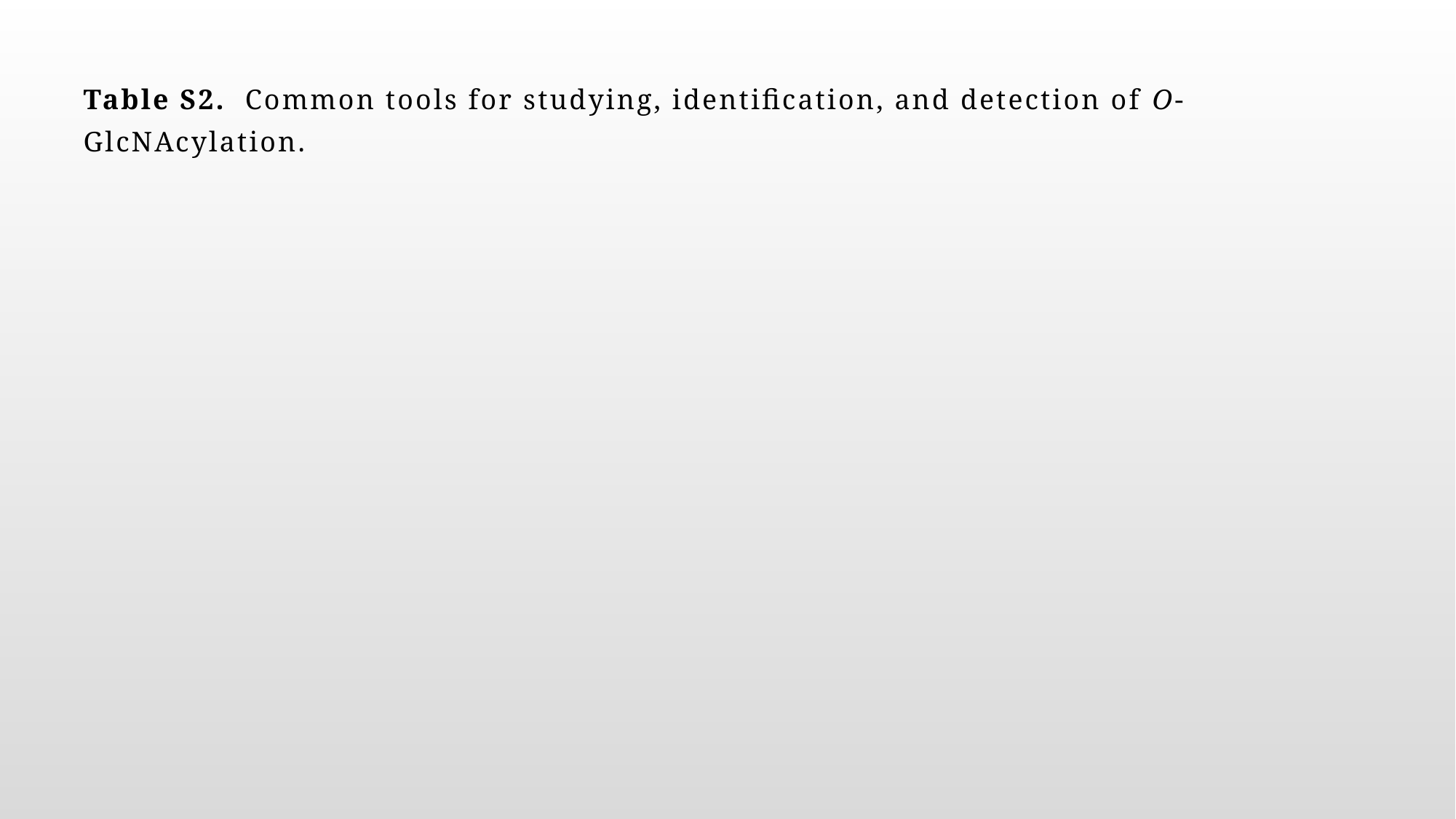

Table S2.  Common tools for studying, identification, and detection of O-GlcNAcylation.

## Slide 4
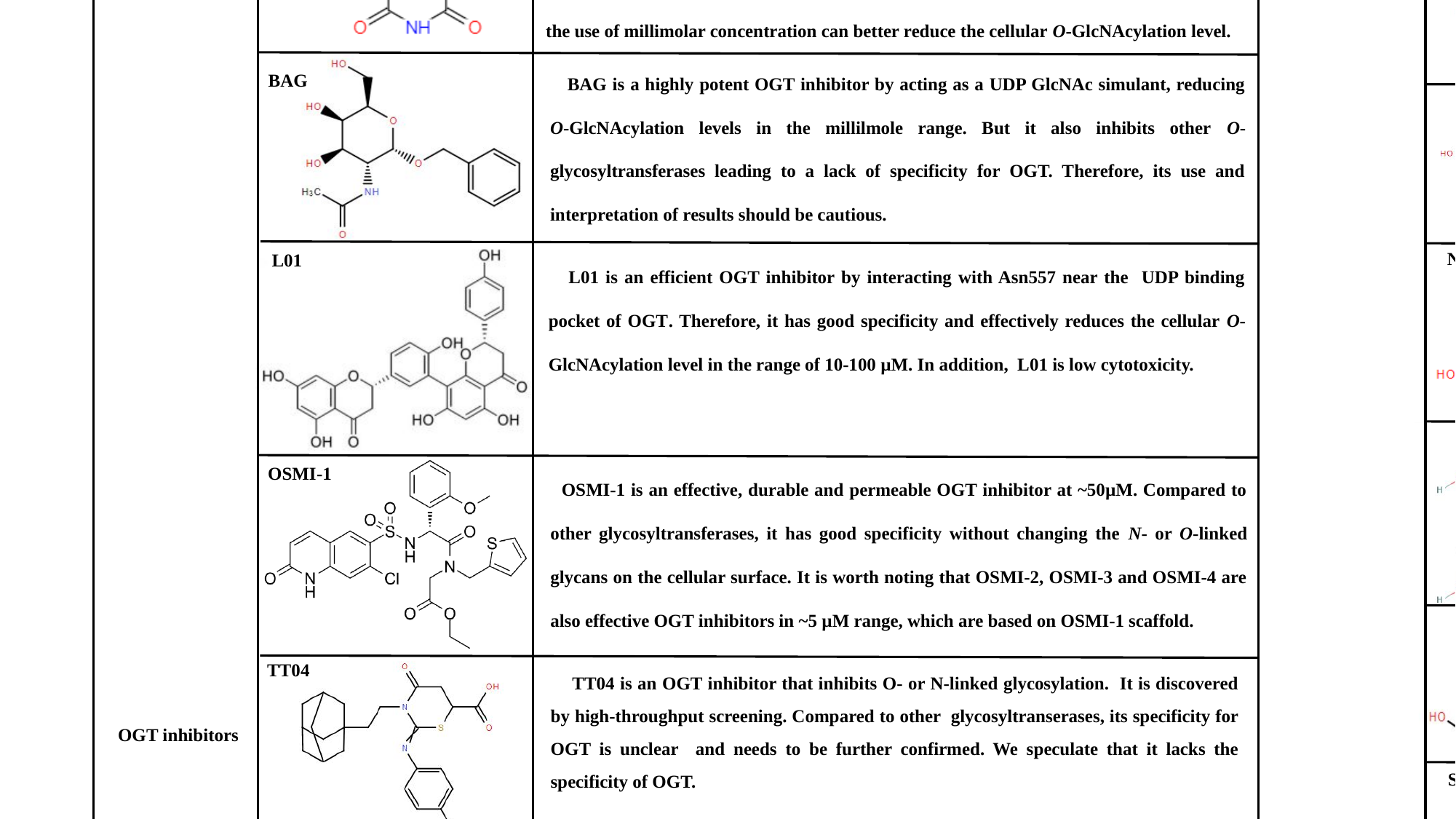

Type
Name
Description
Type
Name
Description
Azaserine
α-GlcNAc Thiolsulfonate
 Azaserine is a glutamine analogue that reduces HBP flux by inhibiting GFAT; However, it also inhibits other glutamine pathways, leading to its lack of specificity, which requires careful interpretation of its results. It continues to be widely used in the absence of any other small molecule GFAT inhibitors.
 Alpha-GlcNAc thiolsulfonate is a highly efficient short OGA inhibitor (Ki = 10 μM) , and showed some selectivity for long OGA and lysosomal hexosaminidases.
GFAT inhibitors
DON
GlcNAcstatin
 DON's description is the same as Azaserine.
 GlcNAcstatin is a picomolar O-GlcNAcase inhibitor with the high degree of potency and selectivity.
OGA inhibitors
 Alloxan is a widely used OGT inhibitor. However, it has a certain off target effect, which weakens the inhibitory effect. In addition, Alloxan is cellular toxicity. Therefore, the use of millimolar concentration can better reduce the cellular O-GlcNAcylation level.
Alloxan
Gluco-Nagstatin
 Gluco-Nagstatin is a highly effective OGA inhibitor that based on the natural product Nagstatin. However, Nagstatin is a potent inhibitor of β -hexosaminase. Therefore, it can inhibit OGA, but is more effective against β -hexosaminase.
 BAG is a highly potent OGT inhibitor by acting as a UDP GlcNAc simulant, reducing O-GlcNAcylation levels in the millilmole range. But it also inhibits other O-glycosyltransferases leading to a lack of specificity for OGT. Therefore, its use and interpretation of results should be cautious.
BAG
 PUGNAc is the most widely used OGA inhibitor. Its cell permeability is very good, but its water solubility is limited. It usually needs to be dissolved in DMSO and then diluted into aqueous solution for biological research. The limitation of PUGNAc is that its inhibitory effect on lysosomal hexosamidase is almost the same as that on OGA.
PUGNAc
 L01 is an efficient OGT inhibitor by interacting with Asn557 near the UDP binding pocket of OGT. Therefore, it has good specificity and effectively reduces the cellular O-GlcNAcylation level in the range of 10-100 µM. In addition, L01 is low cytotoxicity.
NAG-thiazoline
L01
 NAG-thiazolin is a competitive OGA inhibitor with higher selectivity compared with other hexosaminidases.
NButGT
 NButGT is an effective OGA inhibitor based on NAG-thiazoline scaffold. But it is less potent than NAG-thiazoline, but more selective than other hexosaminidases.
 OSMI-1 is an effective, durable and permeable OGT inhibitor at ~50µM. Compared to other glycosyltransferases, it has good specificity without changing the N- or O-linked glycans on the cellular surface. It is worth noting that OSMI-2, OSMI-3 and OSMI-4 are also effective OGT inhibitors in ~5 µM range, which are based on OSMI-1 scaffold.
OSMI-1
 Thiamet-G is a widely used OGA inhibitor. It is a derivative of NButGT and has higher stability, water solubility and oral availability.
Thiamet-G
TT04
 TT04 is an OGT inhibitor that inhibits O- or N-linked glycosylation. It is discovered by high-throughput screening. Compared to other glycosyltranserases, its specificity for OGT is unclear and needs to be further confirmed. We speculate that it lacks the specificity of OGT.
OGT inhibitors
STZ
 STZ is a natural product of GlcNAc analogue and is considered to weakly inhibit OGA. However, its inhibitory effect is controversial due to selective toxicity to islet β cells.
ST060266's description is the same as TT40.
ST060266
 Agrocybe aegerita GlcNAc-specific lectin, also known as AAL2 and AANL, is a useful tool for enriching and identifying O-GlcNAcylated proteins and peptides.
Agrocybe aegerita
GlcNAc-specific lectin
Click-IT™ O-GlcNAc
Enzymatic Labeling System
 The Click-iT™ O-GlcNAc Enzymatic Labeling System provides a highly sensitive and efficientmethod for the in vitro modification of O-GlcNAcylated proteins.
ST078925's description is the same as TT40.
ST078925
 Identification
Galactosyl transferase/
[3H]-galactose
 Results in incorporation of 3H into terminal GlcNAc residues on proteins allowing for detection by autoradiography. Can be very time intensive due to low sensitivity of 3H.
 WGA and sWGA are used for immunoblotting. WGA identifies all terminal GlcNAc residues as well as sialic acid. sWGA reduces affinity for sialic acid.
Wheat germ agglutinin (WGA)/
succinylated WGA (sWGA)
β-elimination followed by michael addition of dithiothreitol
 It relies on the β-elimination of phosphate or O-GlcNAc under basic conditions followed by Michael addition using dithiothreitol or a biotinthiol probe.
UDP-5S-GlcNAc
 UDP-5S-GlcNAc's description is the same as BAG. UDP-5S-GlcNAc is not cell-permeable and therefore were effective in vitro. The peracetylated form of 5S-GlcNAc crosses cell membrane and converted to UDP-5S-GlcNAc, which binds to active site of OGTand reduces O-GlcNAcylation levels in cells in the 10-100 µM range. The inhibition of OGT by 5S-GlcNHex also has a similar mechanism.
 It replaces O-GlcNAc with GalNAz, combined with a biotin or streptavidin cleavable linker can be used to enrich O-GlcNAcylated peptides.
Chemi-enzymatic labeling
 Even the most widely used antibodies perform poorly in immunopurification. Therefore, its application needs to be cautious.
Purification
Immunoprecipitation
 This is a relatively simple enrichment method. But pretreatment is needed to remove N/O-linked glycans to avoid co-purification of other glycoproteins for high specificity.
WGA-agarose
 BZX is a cell permeable, irreversible and covalent inhibitor of OGT that compete with the nucleotidesugar donor. Its structural feature is that it contains benzoxazolinone core. However, it was shown to be potentially toxic and have off-target effects.
BZX
 The most widely used and commercialized O-GlcNAc antibodies are listed here, including CTD110.6, RL2, HGAC39, HGAC85, MY95, 1F5.D6, 9D1.E4 and 18B10.C7.
O-GlcNAc antibodies
 The most widely used and commercialized OGT antibodies are listed here, including D1D8Q, HCFC1,EPR12713 and EPR12712.
OGT antibodies
Antibody
 The most widely used and commercialized OGT antibodies are listed here, including EPR7154(B), EPR21929-223 and EPR21929-274.
OGA antibodies
